# Non-Random mtDNA Segregation Patterns Indicate a Metastable Heteroplasmic Segregation Unit in m.3243A>G Cybrid Cells

**DOI:** 10.1371/journal.pone.0052080

**Published:** 2012-12-18

**Authors:** Anton K. Raap, Roshan S. Jahangir Tafrechi, Frans M. van de Rijke, Angela Pyle, Carolina Wählby, Karoly Szuhai, Raimond B. G. Ravelli, René F. M. de Coo, Harsha K. Rajasimha, Mats Nilsson, Patrick F. Chinnery, David C. Samuels, George M. C. Janssen

**Affiliations:** 1 Department of Molecular Cell Biology, Leiden University Medical Centre, Leiden, The Netherlands; 2 Wellcome Trust Centre for Mitochondrial Research, Newcastle University, Newcastle upon Tyne, United Kingdom; 3 Imaging Platform, Broad Institute of MIT and Harvard, Cambridge, Massachusetts, United States of America; 4 Centre for Image Analysis, Uppsala University, Uppsala, Sweden; 5 Department of Neurology, Erasmus Medical Center, Rotterdam, The Netherlands; 6 Center for Human Genetics Research, Vanderbilt University Medical Center, Nashville, Tennessee, United States of America; 7 Department of Genetics and Pathology, Uppsala University, Uppsala, Sweden; University of Texas Health Science Center at San Antonio, United States of America

## Abstract

Many pathogenic mitochondrial DNA mutations are heteroplasmic, with a mixture of mutated and wild-type mtDNA present within individual cells. The severity and extent of the clinical phenotype is largely due to the distribution of mutated molecules between cells in different tissues, but mechanisms underpinning segregation are not fully understood. To facilitate mtDNA segregation studies we developed assays that measure m.3243A>G point mutation loads directly in hundreds of individual cells to determine the mechanisms of segregation over time. In the first study of this size, we observed a number of discrete shifts in cellular heteroplasmy between periods of stable heteroplasmy. The observed patterns could not be parsimoniously explained by random mitotic drift of individual mtDNAs. Instead, a genetically metastable, heteroplasmic mtDNA segregation unit provides the likely explanation, where stable heteroplasmy is maintained through the faithful replication of segregating units with a fixed wild-type/m.3243A>G mutant ratio, and shifts occur through the temporary disruption and re-organization of the segregation units. While the nature of the physical equivalent of the segregation unit remains uncertain, the factors regulating its organization are of major importance for the pathogenesis of mtDNA diseases.

## Introduction

Mammalian cells contain thousands of copies of mitochondrial DNA (mtDNA) which replicate independent of the nuclear DNA synthesis phase. At mitosis, mtDNAs are generally thought to be partitioned randomly between daughter cells [1]. If a cell contains a mixture of mutated and wild-type mtDNA, a situation referred to as heteroplasmy, then this process leads to subtle differences in the proportion of the different genomes in daughter cells through vegetative segregation. In time, this process can lead to the accumulation of the mutant species in some daughter cells, and the loss of mutation from others.

Mitochondrial diseases caused by point mutations of mtDNA are characterized by clinical heterogeneity, as exemplified by the most common pathogenic heteroplasmic variant m.3243A>G in *MTTL1* coding for mitochondrial tRNA^Leu(UUR)^. M.3243A>G causes the maternally inherited diabetes and deafness (MIDD; MIM 520000) [2], [3], mitochondrial myopathy encephalopathy lactic acidosis and stroke-like episodes (MELAS; MIM 540000) [4], and a number of other clinical phenotypes [5]. Segregation to high levels of mutated mtDNA is the principal factor in determining the pattern and severity of the disease phenotype [6]–[8], but the underlying mechanisms of segregation are poorly understood.

In recent years, it has become increasingly clear that mammalian mtDNA is organized in nucleoprotein complexes commonly referred to as nucleoids [9]. Knowledge of the protein composition and dynamics of mammalian nucleoids is steadily increasing [10]–[16]. While of basic interest, such knowledge is essential for a complete understanding of the pathogenesis of inherited mtDNA diseases [17]–[21]. A number of physical studies reported the average number of mtDNA copies per nucleoid at between 2 and 10 molecules in mammalian cells [22]–[24], raising the possibility that a nucleoid could contain mixed species of mtDNA (heteroplasmy), and thus play a role in mitotic segregation of different mtDNA genotypes [25]. On the other hand, recent super-resolution STED microscopy analysis [26] reported ∼1.4 mtDNA molecules per nucleoid, suggesting that the smallest inheritable or segregation unit is a single molecule of mtDNA [26]. In contrast, cell population segregation studies, complementation studies and the threshold effect all indicate a larger segregation unit with at least 5–10 copies of mtDNA [24], [27]–[32]. These different explanations are not mutually exclusive, with the various different scenarios dominating the picture at different stages of the cell life-cycle and in different tissue types. However, there have been few studies tackling this important issue in an experimental system on a large scale, and at different time points.

Transmitochondrial cybrids provide valuable sources for *in vitro* experimental mitotic segregation analysis. They are created by fusion of enucleated cells carrying two different mtDNA sequence variants with nucleated cells that have no mtDNA (ρ^0^ cells) [33]. Two patterns of heteroplasmy evolution have been found for m.3243A>G clones cultured under non-selective conditions, stable heteroplasmy and heteroplasmy shifts to either wild-type or mutant [29], [34] (for a review see [35]). These observations are largely based on the average mutation level measured in bulk DNA extracted from cellular homogenates of cybrid clones at different passages, and are explained primarily in terms of random mitotic mtDNA segregation in combination with cellular selection and replicative advantages of mtDNA genotypes [29], [34], [35].

However, stability of bulk mtDNA mutation loads during culture could also result from constraints of mtDNA segregation. Only single cell analysis can make the distinction. It is in this context that Lehtinen *et al* [27] embarked on a single cell m.3243A>G segregation study, cloning single cells by limiting dilution and using last hot cycle PCR-RFLP (Restriction Fragment Length Polymorphism) after expansion of the clone to measure the mutation loads of individual cells in early and late passages (∼30 weeks). As a consequence of the low cloning efficiencies, only tens of cells of a sample could be measured. For m.3243A>G clones with or without the m.12300 suppressor mutation [36] the distribution of single cell mutation loads showed negligible change in variance, with mean heteroplasmy levels remaining constant. Mathematically modelling the process of random segregation, and assuming single mtDNAs constitute the independent segregation units, the authors concluded that these distributions could be explained by random segregation of mtDNAs if the cellular mtDNA content was >1000 mtDNAs/cell [27]. The absolute cellular mtDNA copy number was not measured, but a value of ∼1000–2000 is within the range reported for several 143B cultures [22]–[24] (and this study). However, subsequent clones displayed discrete shifts in heteroplasmy, which could not be attributed to random drift, leading to the suggestion that segregation and selection only occurred *after* a relaxation of a pre-existing segregation constraint. In other words, the segregation of mtDNA genotypes is generally suppressed as a rule, but occasionally this suppression is transiently lost, leading to shifts in mtDNA heteroplasmy. Nucleoid mitosis was proposed to confer the segregation constraint [25].

**Figure 1:**
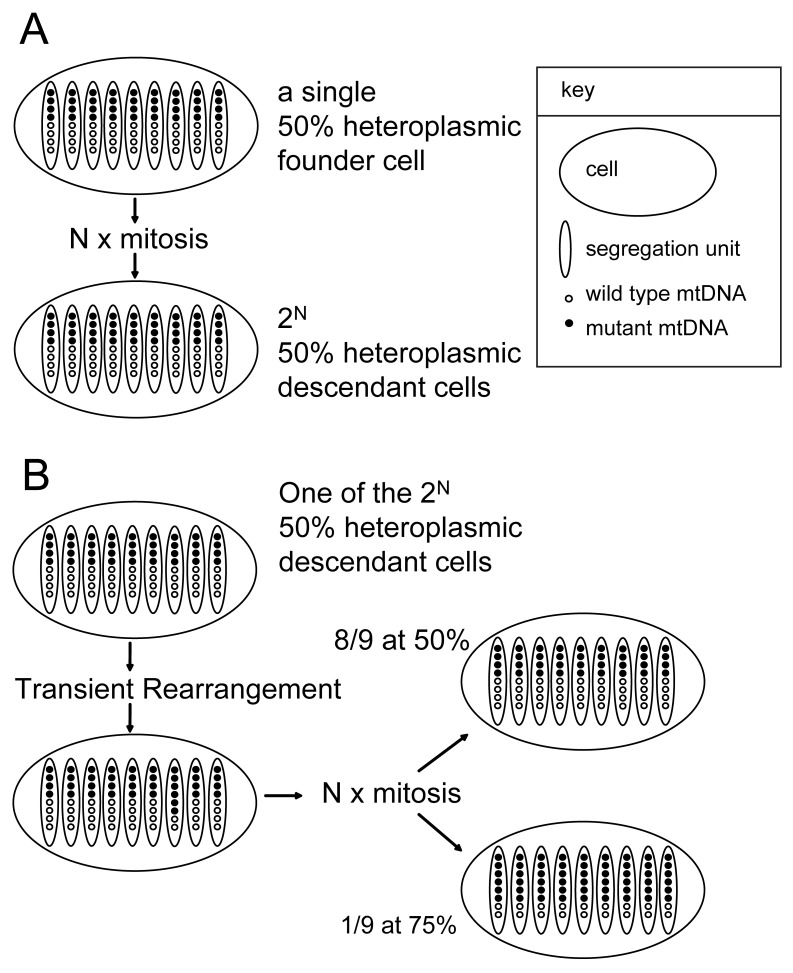
The metastable-segregation-unit working model. (A) Schematic representation of the mitotic consequences of a stable heteroplasmic mtDNA segregation unit. The hypothetical founding cell is 50% heteroplasmic with 72 mtDNA molecules arranged in 9 segregation units each with 8 mtDNA’s, 4 of which are variant. Upon their faithful replication and partitioning to daughter cells for N mitosis, all 2^N^ descendant cells acquire the same 50% heteroplasmy level. (B) Schematic representation of the mitotic consequences after a metastable event in a cell from (A) such that one of its units has 6 instead of 4 variant mtDNA molecules. Upon random mitotic segregation of the units, 1/9^th^ of the cells will become fixed at 75% heteroplasmy and 8/9^th^ at 50%. Other scenarios can be envisaged. For example, a random redistribution of all mtDNAs in a cell from (A) will result in units with 0/8^th^, 1/8^th^ ….7/8^th^, 8/8^th^ mutation loads with binomially distributed frequencies. For simplicity, a uniform heteroplasmic segregation unit with identical number of copies of mtDNA is depicted. Slight variation in mtDNA copy number per unit in a cell will rapidly evolve through random mitotic segregation of these units into multiple cell types with each another uniform heteroplasmic segregation unit (like in B).

In view of the fundamental importance of the mtDNA segregation process, it is remarkable that few studies have advanced our understanding of this process, which still remains uncertain. Only one study [37] provided further evidence based on one m.3243A>G clone showing constrained mtDNA segregation throughout many mitoses. This paucity of experimental data likely relates to the limited throughput of the assay used and the low sub-cloning efficiencies encountered. To overcome these problems and to test hypotheses of mitotic mtDNA segregation, we developed methods for directly quantifying m.3243A>G mutation loads in single cells, allowing unbiased analysis of hundreds of cells per sample [38]–[40] (and Figures S1 and S2), and allowing us to provide a comprehensive view on heteroplasmy evolution in progeny of heteroplasmic m.3243A>G cells through consecutive passages from a single cell founder. These observations cast light on the underlying mechanism of mtDNA segregation, and led to the proposition of a ‘metastable-segregation-unit’ working model as depicted in Figure 1.

## Materials and Methods

### m.3243A>G cybrid Clones

The m.3243A>G transmitochondrial 143B cybrid cells (V and GB from MIDD; G from MELAS) used in this study have been previously established [38], [39], [41]. Cybrid cloning was by limiting dilution or single cell flow sorting as indicated in the text. Cybrid cells were grown on non-selective Dulbecco’s Modified Eagle’s medium containing high glucose (4.5 mg/ml) and 110 µg/ml pyruvate supplemented with 50 µg/ml uridine and 10% foetal bovine serum. After the first outgrowth to near-confluence (∼a million cells corresponding to ∼20 population doublings) in 9 cm dishes, they were cultured with two passages per week. The split was 10% for most clones, but for a few it was adapted to 5% or 15% so as to reach confluence before the next passage. Cultures were frequently inspected to prevent over-confluence or acidification of the medium. Passages were archived in liquid nitrogen every two to four weeks. For determination of the average mutation load, bulk DNA samples were prepared at least every two weeks.

### Single Cell m.3243A>G Mutation Load Assays

For the determination of single cell m.3243A>G mutation loads, two independent methods were used. One is based on PCR-RFLP of single sorted cells, employing melting temperature characteristics (referred to as PCR-RFMT) of the fragments using SYBR Green as reporter [38]. The second is an m.3243A>G in situ genotyping method based on Padlock probe hybridization and Rolling Circle Amplification (Padlock/RCA) [39] in combination with image analysis to count the number of detection events (dots) per cell of m.3243A (green) and m.3243G (red) mtDNA and derive the mutation load as the red/red+green ratio [40]. As shown earlier [38] and here, single cell heteroplasmy results obtained with both methods are in accord. With single cell PCR/RFMT standard deviations (SD’s) of 4–8% heteroplasmy are obtained with ∼250 cells analyzed [38]. As shown in Figure S1, with the Padlock/RCA method similar accuracies are obtained.

### Bulk mtDNA Mutation Load Assay

Average cellular m.3243A>G mutation load on bulk DNA was determined by PCR/RFLP [8] or PCR/RFMT in triplicate with SD’s typically being ∼1% [38], [42].

### mtDNA Copy Number Determination

Average cellular mtDNA copy number was determined on bulk DNA with the aid of the ΔCt method [43] using two globin genes per cell after having established disomy for chromosome 11 of the 143B nuclear genome by molecular karyotyping [44]. The SybrGreen Master mix (Applied Biosystems, USA) for real-time PCR with primers for β-globin and mtDNA as well as PCR conditions were as described [43]. At least four dilutions per sample were used in triplicate. The log dilution *vs* C_t_ values for mtDNA and for globin gene displayed equal slope, and linear correlation coefficients of 0.99 or higher.

### Array Comparative Genome Hybridization

Array comparative genome analysis (Array-CGH) was performed at 1 Mb resolution with a BAC/PAC array as described [45].

### Simulation of Random Mitotic Segregation

Computer simulations of random segregation were started with a single cell containing a given fraction of mutant mtDNA molecules. The mtDNAs in the simulation were copied with no preference to either wild-type or mutant. For simulation details see [46], [47]. The simulated cells were divided and the mtDNA molecules were individually randomly distributed to the two daughter cells. After the simulated cell population has reached 1 million cells we began simulating cell culture passages by selecting 100,000 cells (1/10th of the population) at random and dividing them till 1 million cells was reached.

## Results

### Identification of a Shifting Clone and Single Cell Point Mutation Analyses of its Passages

**Figure 2:**
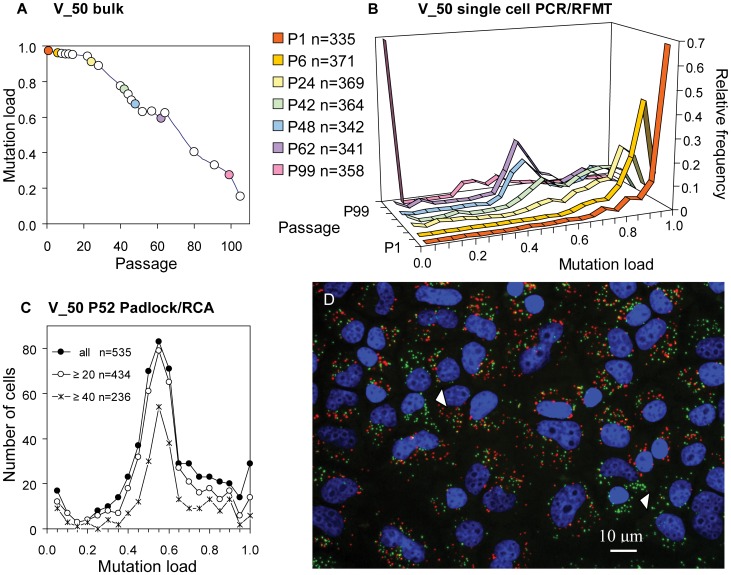
Heteroplasmy evolution of cybrid clone V_50. (A) Average cellular mutation load decreases with increasing passage number of clone V_50. Conventional gel-based PCR-RFLP was used for quantitation of the m.3243A>G heteroplasmy. (B) Relative mutation load PCR-RFMT histograms of flow sorted single cells of selected passages of clone V_50. n refers to the number of single cells evaluated in the histogram. (C) Frequency histograms of m.3243A>G Padlock/RCA mutation loads of cells in V_50 P52 at increasing stringency of the dots/cell number criterion, cells with >0 (all), ≥20 and ≥40 dots/cell. See Figure S1 for choice of stringency and bin size. (D) Microscopic image of V_50 P52 after m.3243A>G Padlock/RCA *in situ* genotyping. Note in this microscopic field the presence of homoplasmic wild type cells (arrow heads) amidst the heteroplasmic cells. See Figure S2 for discussion on the number and nature of the signals.

The m.3243A>G 143B cybrid clone V_50 was originally identified as a homoplasmic mutant clone. It was monitored for mutation load using bulk DNA and apparently homoplasmic mutant for several months [38], [39], [41]. At a given passage (referred to here as P1) it was found to possess a small fraction of wild-type mtDNA, but drifted to near 100% wild-type over one year (Figure 2A). This observation prompted us to develop the single cell m.3243A>G mutation load assays to measure this and other shifting clones, to determine whether heteroplasmy evolution at the cellular level was compatible with random mitotic segregation of individual mtDNA molecules, or with the metastable-segregation-unit working model.

When analysed by single cell PCR-RFMT (PCR-RFLP employing Melting Temperature characteristics) [38], individual cells of the first V_50 passage were mostly 95–100% mutant type (Figure 2B) with a few cells containing lower levels of m.3243A>G. At P6 the majority of cells had 90–95% heteroplasmy. At passage 24 the cells with 90–95% mutation load still dominated the histogram. In the three next passages analysed, a main peak at 50–55% heteroplasmy emerged, while at P99 a main peak at 0–5% was evident. The presence of cells with stable 50–55% heteroplasmy in the passage range 42–62 was confirmed qualitatively and quantitatively by in situ genotyping method based on Padlock probe hybridization and Rolling Circle Amplification (Padlock/RCA) analysis [39] of cells at passage 52 (see Figure S1 for dots/cell stringency considerations). Using both methods, the results showed the presence of the significant subpopulation of cells in the 50–55% range (Figures 2C and 2D). We inferred, therefore, that V_50 cells with newly acquired 50–55% mutation loads had restricted mtDNA segregation, and presumably a selective growth advantages to enable us to observe them as major peaks in the distribution of cellular heteroplasmy levels. We did not observe extensive co-localisation of mutated (red) and wild-type (green) signals using Padlock/RCA FISH techniques (Figure 2D). Although low hybridization efficiencies make it on theoretical grounds improbable to see many ‘yellow’ signals (Figure S2), the Padlock/RCA efficiencies are suitable for quantitation of single cell mutation loads, but insufficient for assessment of the genotypic status of individual heteroplasmic segregation units.

### Selection and Single Cell Analysis of Additional Shifting Clones

**Figure 3:**
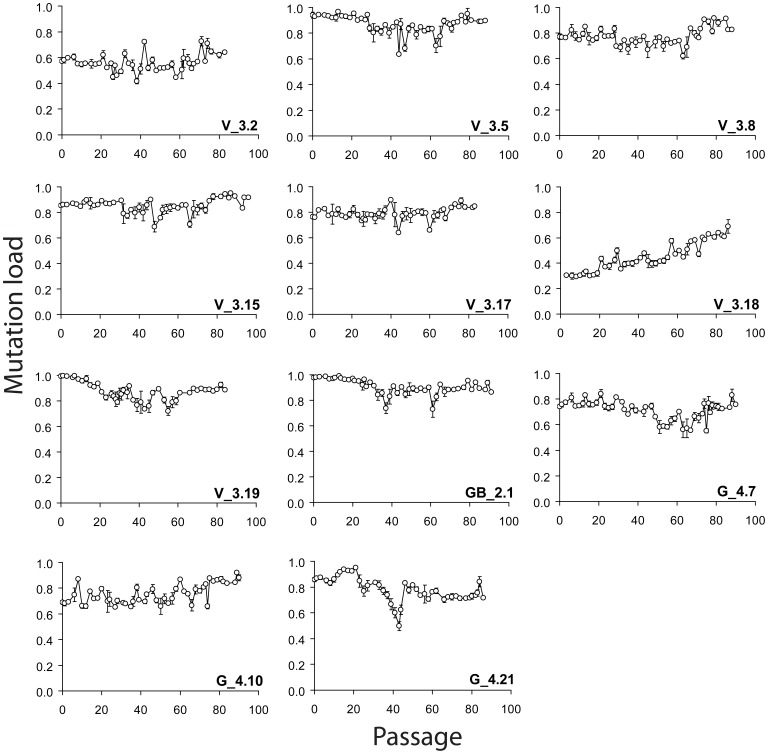
Evolution of bulk mtDNA mutation load during long term continuous culture of 11 cybrid clones. Average m.3243A>G mutation loads were determined with PCR/RFMT on bulk DNA in triplicate. The size of the data marker masks error bars of most data points.

Based on these findings, we postulated a metastable-segregation-unit working model (Figure 1), where cells with a novel heteroplasmy level are generated through the reorganization of the segregation unit, before replicating faithfully. To substantiate the model experimentally, we flow sorted single cells from V_50 P48 and studied mtDNA segregation in the sub-clones. Seven V_50 P48 sub-clones, one GB and three G sub-clones were cultured continuously for 80–90 passages and analyzed by bulk mutation load measurements (Figure 3). We observed minor fluctuations in the average heteroplasmy level in most sub-clones, however, G_4.21 and V_3.18 showed considerable shifts (>25–30%) during this period. G_4.21 shifted towards wild type and then rapidly back to mutant, while V_3.18 showed a steady shift from wild type towards mutant. Thus, stable heteroplasmy appears to be the rule. On the same 143B nuclear background, heteroplasmy can also shift towards either wild type or m.3243A>G mutant within a single clone.

**Figure 4:**
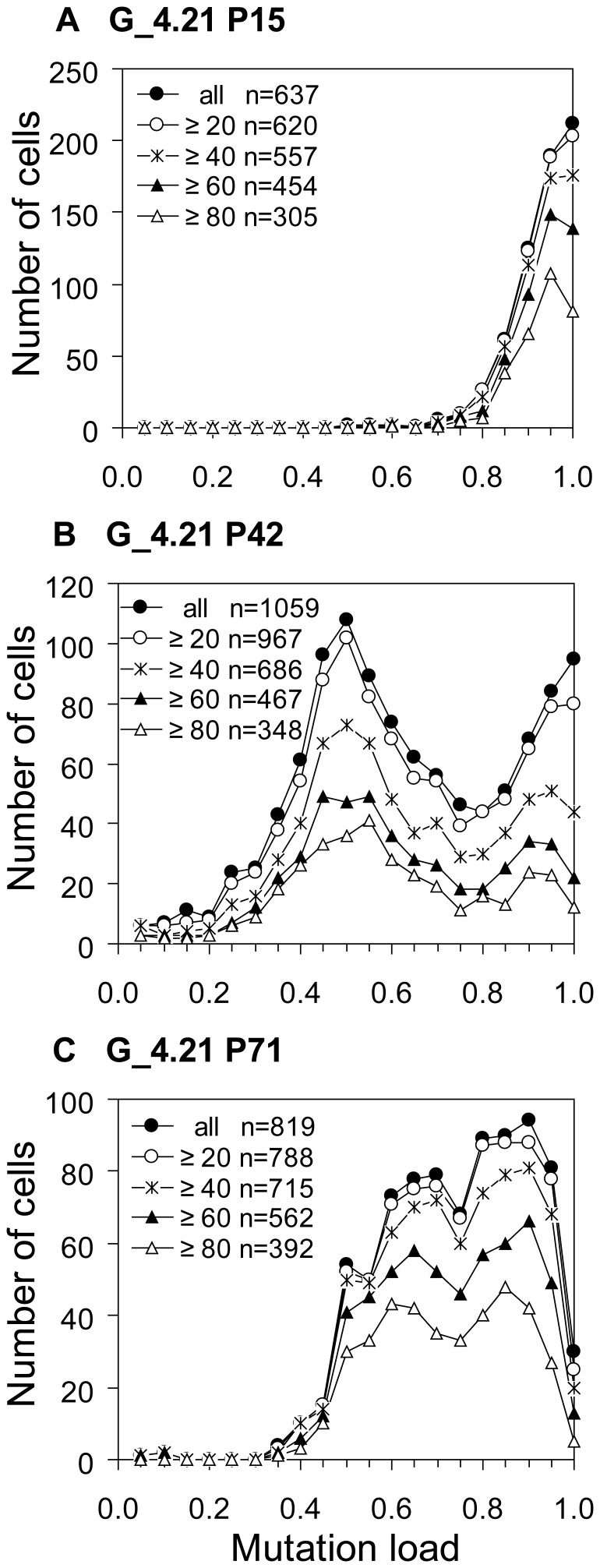
Cellular heteroplasmy evolution of clone G_4.21 by Padlock/RCA analysis. The m.3243A>G mutation load of single cells was determined by Padlock/RCA in passages 15 (A), 42 (B) and 71 (C). Dots/cell number may reflect detection efficiency, but also the actual segregation unit number. Since detection efficiency is arguable low (Supplemental Figure S2), in the order ∼5%, cells with a wide range of dots/cell number will be observed, independent of true variations in segregation unit number. The frequency histograms of the three passages are shown separately at increasing stringency of the dots/cell criterion (>0 (all) up to ≥80 dots/cell) to emphasize that the peaks and shoulders are genuine. Thus, the outcome of the experiment does not change substantially with the smaller amount of cells with larger dots/cell (≥60 or ≥80), which makes calculation of mutation load more solid (See also Figure S1 for choice of stringency and bin size). Due to the choice for a 5% bin, it is not apparent that the cells in the 95–100% bin of P15 are for the very great majority heteroplasmic: in that bin, 94% of the cells with ≥40 dots/cell and 92% of the cells ≥80 dots/cell bin had in fact one or more green (wild type mtDNA) present.

**Figure 5:**
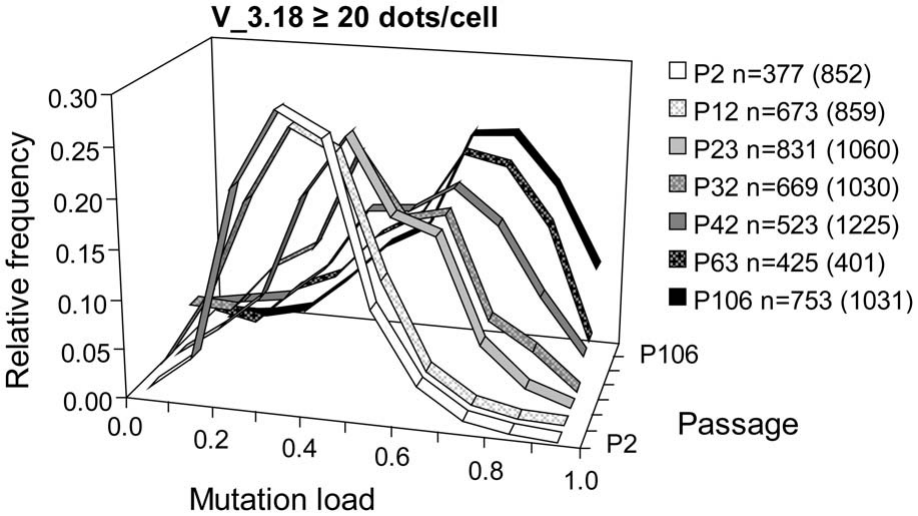
Cellular heteroplasmy evolution of V_50 P48 sub-clone V_3.18 by Padlock/RCA analysis. m.3243A>G mutation load of single cells of the sub-clone V_3.18 derived from V_50 P48 was assessed with Padlock/RCA. Relative frequency histograms of cells in 7 passages are shown. Since 3 of the 7 passages did not meet the criterion of >30% of the cells having ≥40 dots/cell (see Figure S1), the relative frequency histograms are therefore shown at ≥20 dots/cell stringency and with 10% bin. n refers to the number of single cells evaluated in the histogram whereas numbers in parentheses represent the total number of cells analyzed.

To provide further confirmation, several passages of G_4.21 and V_3.18 were analysed by single cell Padlock/RCA (Figure 4). At P15 of G_4.21, the vast majority of cells had high mutation loads (90–95%), while in P42 an additional prominent 45–50% heteroplasmy subpopulation was present. At P71, this 45–50% heteroplasmy subpopulation was still present, but reduced in size as shown by the “distribution shoulder” at 45–50% heteroplasmy. Next to the high heteroplasmy subpopulation, a new significant subpopulation of cells with 65–70% heteroplasmy was evident at this passage. Overall, these results show that multiple distinct and semi-discrete heteroplasmic cell subpopulations had arisen from the founding cell of this clone. For V_3.18, the shift to a higher average mutant load appeared to be due to a decrease in relative frequency of cells with the lower mutation levels, and an increase in frequency of cells with the higher heteroplasmy (Figure 5). This indicates that during culture, cells emerged with a higher, stable mutant load that gained growth advantage.

### Nuclear Genomic Instability and Growth Advantage

**Figure 6:**
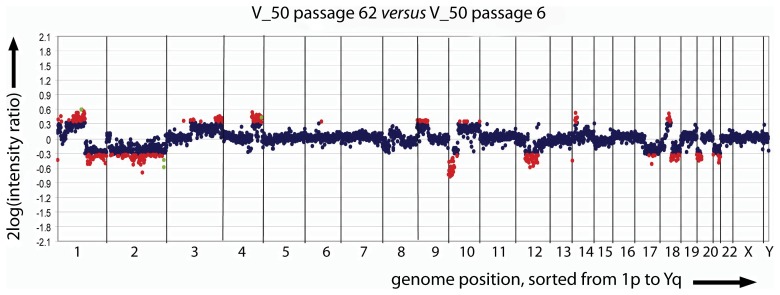
Genomic instability of the 143B host nucleus as revealed by array-CGH. Array-CGH was at 1 Mb resolution with a BAC/PAC array using genomic DNAs from V_50 P62 and V_50 P6 as test DNA and reference DNA respectively. Red, blue and green dots represent, respectively, copy number change, no copy number change and natural copy number variations. Numbers on the X-axis represent human chromosome numbers.

The transient release of segregation constraints provided an explanation for the emergence of cells with altered, but stable heteroplasmy [27]. However, for these cells to become apparent in the single cell mutation load histogram, they must have gained growth advantage. The fact that cellular heteroplasmy shifts to mutant type (V_3.18), to wild type (V_50) as well as back and forth (G_4.21), indicates that cellular growth advantages are not strictly coupled to the level of heteroplasmy *per se*. For example, in P71 of G_4.21, the cells with the higher heteroplasmy levels of 65–70% and 90–95% have “out-competed” the 45–50% heteroplasmy subpopulation that is prominently present in P42. To determine whether gross changes in nuclear DNA composition correlated with these shifts, we performed array-CGH [45] using genomic DNA from V_50 passage 6 and 62. This showed the nuclear genome of the 143B host nucleus to be genetically unstable (Figure 6), raising the possibility that disruption or duplication of nuclear genes were responsible for growth advantage of a cell that happened to host an altered mtDNA segregation unit so that it became apparent in the cell population.

### Suppression of Random Mitotic mtDNA Segregation

**Figure 7:**
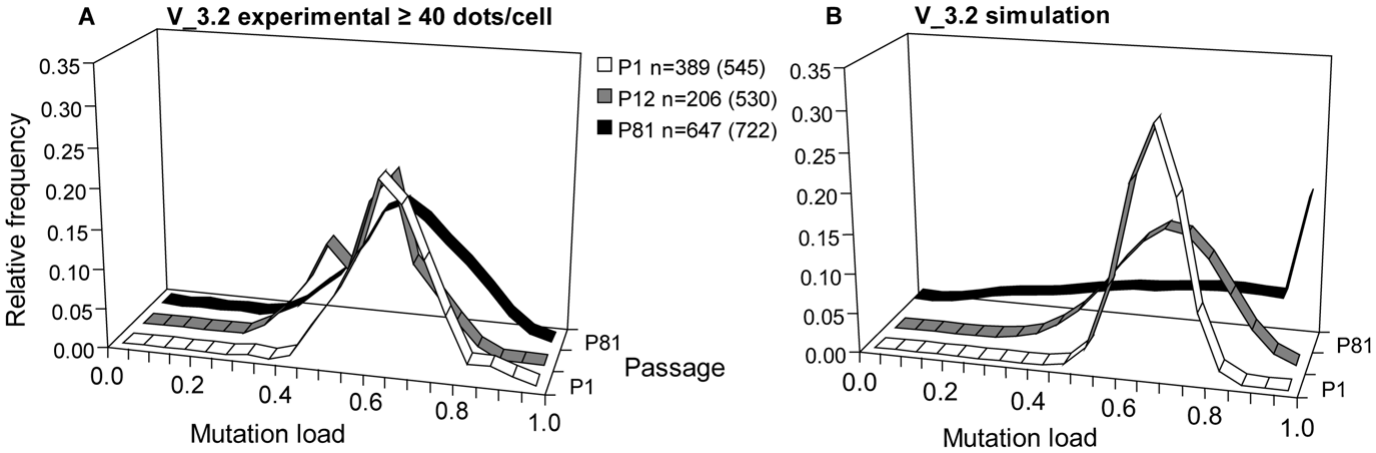
Cellular heteroplasmy evolution of V_50 P48 sub-clone V_3.2. (A) Relative m.3243A>G Padlock/RCA mutation load frequency histograms of cells in passages 1, 12 and 81 of sub-clone V_3.2. Histograms from cells with ≥60 dots/cell had similar shapes. Numbers in parentheses represent the total number of cells analyzed. (B) Relative mutation load frequency histograms of cells in passages 1, 12 and 81 of V_50 P42 sub-clone V_3.2 generated by computer simulation of random segregation (mtDNA copy number input 1,800/cell). Additional computer simulations showed that an mtDNA copy number input of 12,000 is required to explain the experimental distribution at P81 by random mtDNA segregation.

The presence of cell populations with distinct and stable heteroplasmy levels developing from three different clones strongly suggests that there are mtDNA segregation constraints in action at different time points. To determine whether the metastable-segregation-unit could explain this we studied individual cells in clone V_3.2, which had been shown to have stable heteroplasmy over ∼80 passages (Figure 3). When analysed at the single cell level by Padlock/RCA, a prominent peak was found at 60–65% mutation load in P1, P12 and P81 (Figure 7A). V_3.2 contained on average ∼1,800 mtDNAs per cell. Using the average heteroplasmy of P1 (67%) as founding cell heteroplasmy and 2,000 as mtDNA copy number within the cell, we carried out an *in silico* simulation of the random mitotic segregation of mtDNA variants within dividing cells, and compared the simulation results to the experimental data (Figure 7B). The simulations predicted much greater variation than we observed experimentally. Computer simulations showed that the relatively stable distribution of heteroplasmy from P1 through P81 could only be consistent with random segregation of mtDNAs if the cellular mtDNA content was ∼12,000 mtDNAs/cell. The contradiction with the simulation indicates that the mtDNA molecules are not randomly segregating, but are restricted in clone V_3.2.

**Figure 8:**
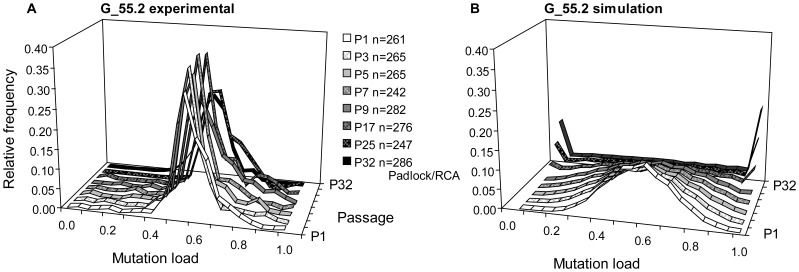
Cellular heteroplasmy evolution of clone G_55.2. (A) Relative m.3243A>G mutation load frequency histograms of cells in multiple passages of G_55.2. With exception of P32, histograms were produced by PCR/RFMT of flow-sorted single cells. Mutation loads of cells in P32 were measured by Padlock/RCA using cells ≥40 dots/cell, which represented 80% of the analyzed cells. SDs for the single cell PCR/RFMT mutation load determinations of P1–P25 ranged from 4 to 8%. (B) Relative mutation load frequency histograms of cells in passages P1–P32 of clone G_55.2 generated by computer simulation of random segregation.

Similar restriction of mtDNA segregation was observed with clone G_55.2. This clone had an average cellular heteroplasmy level of 55% at P1 and a relatively low average copy number of ∼350. Figure 8A shows the experimental single cell PCR-RFMT histograms for several passages unto P25. We also measured mutation loads of P32 of G_55.2 by Padlock/RCA. This distribution was very similar to those of the earlier passages analyzed by PCR/RFMT and is also displayed in Figure 8A. Computer simulations of these G_55.2 passages (Figure 8B) showed that under a random segregation regime at P32 the great majority of cells should have been homoplasmic. >10,000 mtDNAs were required in the computer simulation to approach the shape of the G_55-2 P32 histogram. Our results thus indicate that segregation of mtDNA is also restricted in clone G_55.2.

## Discussion

The most parsimonious explanation for our observations is a metastable segregating unit containing several mtDNA molecules. For the most part, these units replicate faithfully, and are randomly distributed between daughter cells during mitosis. This explains why a particular heteroplasmy level can be stable over many passages. However, the metastable unit re-organizes from time to time. It is this re-organization that can cause the dramatic shifts in heteroplasmy that we have observed in otherwise stable m3243A>G cell lines. Thus our model can be considered an extension of the original faithful nucleoid model proposed by Jacobs *et al* [25]. A prediction of the number of mtDNAs per segregation unit can be derived from the spacing between mutation load peaks in the experimental histograms. The present results do not cover the full heteroplasmy range, but the closest spacing found by us was 5–10%, which is in the same range as the ∼8% shifts seen with clones line G5s and G6s by Lehtinen *et al* [27]. This would imply a minimum of 10–20 mtDNAs per segregation unit. We cannot exclude the most extreme case that the segregation unit consists of the entire mtDNA population of the cell [25].

The unstable element of our working model, transient reorganization of the segregation unit (Figure 1B), can explain the appearance of cells with newly acquired distinct and stable mutation loads. Their contribution to the cell population will be small and they would not be readily observable in the histograms. For instance, a single cell in a clonal culture with 5% mutation load (1 mutant mtDNA and 19 wild-type mtDNAs) in all of its say 100 segregation units may undergo a rearrangement in one of its segregation units to 10% mutation load (2 mutant mtDNA and 18 wild-type mtDNAs). This rearranged cell, amidst hundred thousands of cells in the culture, would contain 1 segregation unit with 10% mutation load and 99 segregation units with 5% mutation load. Upon random mitotic segregation of the units, only 1% of the cells originating from the single rearranged cell will become fixed at 10% mutation load, and the remaining 99% of the cells at 5% mutation load. This latter 1% of cells with 10% mutation load amounts far less than 1% in the whole culture, and will not easily attract attention. We therefore inferred that a growth advantage underlies our observation of emergence of significant subpopulation with discretely shifted heteroplasmy (Figures 2B, 2C, 4 and 5). We propose a nuclear, rather than a mitochondrial determined growth advantage, and envisage a scenario in which segregation units with an altered uniform heteroplasmy “hitchhike” with a newly generated nuclear genomic constitution that conferred growth advantage. Therefore, transiently reorganized sub-populations can only be observed in a nuclear genetic instable host like the 143B osteosarcoma.

The observation from bulk mutation load analysis that cybrid cultures can reside for long periods in a near-homoplasmic state (*e.g.* line G [27], [36], V_3.19, GB_2.1 (Figure 3), and V_50 before it entered our single cell analysis) is seemingly not in line with the metastable-segregation-unit model. However, this behaviour can be explained if in the founding cell (V_50 as example) a single segregation unit was present with one wild type mtDNA and say 9 mutant mtDNAs, and that the remainder of the segregation units was uniformly homoplasmic mutant. This 1/10^th^ wild type segregation unit then would segregate to fixation in progeny. With say 100 segregation units per cell this will occur fairly rapid, but the contribution of cells carrying such uniform 1/10^th^ wild type segregation unit to the whole population will maximally reach 1%, thereby explaining the high near-homoplasmic bulk mutant loads with only 0.1% wild type mtDNA. We envision that members of this subpopulation gained growth advantage to constitute a clearly shifted population, e.g. the 90–95% m. 3243A>G cells of V_50 P6. The minor fluctuations in mutation load (Figure 3), if real, may be explained by cells having gained growth advantage in the first place, but for some reason expired and disappeared again from the culture leaving behind the original cell population.

The mitochondrial nucleoid has long been considered as the physical equivalent of the segregation unit [16], [24], [25] and led to the proposition of the faithful nucleoid model: the unit of genetic function comprises a group of mtDNA molecules that are semi-permanently associated as a mitochondrial nucleoid, (heteroplasmic) nucleoids replicate faithfully generating daughter nucleoids that have identical genetic composition to each other and their ‘parent’, daughter nucleoids can themselves segregate freely. The model elegantly accommodates important mitochondrial genetic concepts of 1) the mutation load threshold effect, 2) (non- or limited) genetic complementation and 3) genetic segregation analysis [27] (and this report). Furthermore, it encompasses the notion that mtDNA is less mobile than mitochondrial membranes and that the transcription and translation products of a mtDNA type do not mix with those of another after cybridization [29]–[31]. However, to explain most of these features nucleoids need to be multicopy in nature. Recently, super-resolution STED microscopy analysis revealed that nucleoids frequently contained only a single copy of mtDNA (∼1.4 mtDNA/nucleoid) [26]. Evidently, the genetic segregation unit must be larger than a single nucleoid.

A multicopy segregation unit requires a higher-order structure that prevents exchange of genomes between units. Although this could be composed of protein alone, a membrane-based higher-order structure seems equally attractive, especially when considering the evolutionary origin of mitochondria as a bacterial entity. Remnants of the genome partitioning mechanism on the membrane may still be active and evolved to a multicopy segregation compartment to quell the high mutation frequency of mtDNA. Similar to the bacterial genome, mtDNA is associated to the mitochondrial inner membrane [11]. We speculate that the physical constraint is achieved by a limited spatial domain within the mitochondrial compartment. Compartmentalization is supported by the notion that nucleoids are frequently found in clusters [26]. Analogous to the original faithful nucleoid model, this membranous ‘segregation compartment’ semi-permanently encloses a limited but fixed number of mtDNAs. Such segregation compartments would not easily exchange genomes or their transcription and translation products with other segregation compartments, thereby explaining limited complementation [29]–[31]. After faithful replication of its mtDNA genomes, ‘segregation compartments’ divide in a mitochondrial version of mitosis, yielding two sister segregation compartments with identical genetic composition, which subsequently can freely segregate during cellular mitosis.

Our study contributes to the understanding of mtDNA segregation mechanisms. It establishes that suppression of segregation appears to be the rule and provides a first quantitative underpinning of a model in which uniform heteroplasmic segregation units confer the segregation constraint. Many questions, however, remain unanswered. What is the physical equivalent of the metastable-segregation-unit? What determines the mtDNA copy number of the segregation unit and is it tightly controlled indeed? How does elevated mitochondrial reactive oxygen species production by mtDNA sequence variants modulate segregation [48]? What is the role of mitochondrial fission and fusion processes in biased segregation of mtDNA [49], [50]? How does the concept of separate fusion of outer and inner membranes [51] contribute to the constraints of the ‘segregation compartment’, or may cristae structures sufficiently section the mitochondrial matrix and thereby restrict the mobility of mtDNA and its products? Do Holliday junctions (replication and recombination intermediates linking multiple mtDNA molecules together) contribute to the non-random mtDNA segregation patterns observed in cybrid cells, or is their presence exclusively limited to human heart mitochondria [52]? Are segregation units single or multi-copy in mtDNA in the female germ line during early embryogenesis [53]–[55]?

A recent study suggests that the metastable-segregation-unit model may also hold for other mtDNA mutations. Gilkerson *et al* [24] observed extensive genetic autonomy of nucleoids (corresponding to faithful replication of segregation units) carrying two different mtDNA deletions as well as rare mtDNA exchanges between nucleoids (corresponding to genetic rearrangements of segregation units). Clearly, studies directly testing the metastable-segregation-unit model for other pathogenic mutations and neutral variants are urged for. To resolve mechanisms of clonal accumulation of acquired and inherited mtDNA mutation in aging [56], [57] and mitochondrial disease [17] it will similarly be of importance to get further knowledge of segregation unit organization and dynamics in post-mitotic somatic cells. Segregation suppression in somatic cells may serve to maintain mtDNA genotypic integrity by buffering clonal mtDNA mutation accumulation through random genetic drift, while rearrangements may contribute to mtDNA segregation in early embryogenesis and mtDNA disease development. Finally, a better understanding of the transient reorganization events may provide a therapeutic tool for restoration of mitochondrial function in heteroplasmic cells.

## Supporting Information

Figure S1 **Accuracy of Padlock/RCA.** (A) To assess accuracy of Padlock/RCA, a model system experiment was performed in which we simulated 50% heteroplasmy by hybridizing a bicolour Padlock probe set reporting presence of positions m.2031 and m.12252. The padlock probes for these positions directly flank the Dra1 restriction sites at positions m.2049 and m.12270 from which the Padlock/RCA process is initiated. Padlock probe sequences for positions m.2031 and m.12252 are as follows (italics: probe sequence; underlined: reporter sequence; normal: linker sequence) *TTGTTAGACATGGGG* ATTCCTTTTACGACCTCAATGCACATGTTTGGCTCCTCTTCT*GTTGAGAAAGCCATG* and *ATCTTGGACAACCAGC* ATTCCTTTTACGACCTCAATGCTGCTGCTGTACTACTCTTCT*GTTGAACTAAGATTCT* *.* This probe set was hybridized to cells of passage 1 of clone V_3.2 (V_3.2 P1). Panel (A) shows the ‘mutation load’ distribution in a frequency histogram with a 5% bin for all cells, for cells with ≥20 and ≥40 dots/cell, while the accompanying table gives average ± SD for the mutation load and for the number of dots/cell at increasing stringency of the dots/cell number criterion from >0 (all) up to ≥100 dots/cell. With the average point mutation load being very close to the expected 50% with SD of ∼7% when sampling cells with ≥20 total dots/cell, these data show that with Padlock/RCA similar accuracies as with single cell PCR/RFMT are obtained. Although with the M.3243A>G probe set similar high average dots/cell were obtained with some passages, others gave less. It ranged from 20–88 for all 15 passages analysed with the M.3243A>G probe set, with ∼2-fold variability among passages of a given clone, indicating that procedural aspects affect Padlock/RCA efficiency. In general M.3243A>G mutation load histograms did not change shape significantly when the stringency of the dots/cell was increased, indicating that heteroplasmy histograms can be sampled from sub-populations with the higher dot number. (B) For a passage that yielded intermediate M.3243A>G average dot numbers (V_3.2 P1; average dot number = 59) this is illustrated by showing the minimal effects of excluding cells with <20 and <40 dots/cell on the mutation load histogram and the formal statistics. (C) A similar analysis of the passage with the lowest average dot number in this study (V_3.18 P2; average dot number = 20) illustrates that with such low average total dots/cell numbers, the contribution of cells with >40 dots/cell reduces to <10%. To minimize sampling error, we demanded that more than 30% of the cells contribute to the mutation load histograms. (D) Graph showing the percentage contribution of cells with ≥20 and ≥40 dots/cell as a function of the average dot number per cell. Data from all 15 passages analysed with the M.3243A>G probe set were used. As is evident from the graph, the ‘more than 30% of all cells contribution’ criterion necessitated for 3 of the 15 passages analysed by M.3243A>G Padlock/RCA use of cells with ≥20 dots. In such cases 10% bin histograms were used to present results.
(PDF)

Figure S2 **Efficiency of Padlock/RCA.** Earlier FISH work [58], [59] strongly indicated that detection efficiency (i.e. the fraction of target molecules detected) with small probes (<5000 bp) is low and determined largely by accessibility of the target for detection reagents. In Padlock/RCA FISH a series of in situ enzymatic reaction is involved in the detection, viz. restriction enzyme digestion, 5′-3′ exonuclease, ligation, 3′-5′ nuclease and 5′-3 polymerization. Their cumulative efficiencies will negatively affect overall Padlock/RCA FISH efficiency in the formaldehyde-fixed cells used. If one would e.g. for V_3.2 with its ∼60 dots/cell with the 3243 probe set and ∼1800 mtDNA on average per cell consider a dot as originating from single mtDNAs not organized in any structure then efficiency is apparently only ∼3%. However, in the faithful nucleoid model of Jacobs *et al* [25] as well as in the one proposed here, multiple mtDNAs are presumed to be organized in a limited space. This implies that co-localization of red and green padlock/RCA signals should be seen in heteroplasmic cells, the frequency of which is a function of the mtDNA staining efficiency, the number of mtDNAs per segregation unit and their mutant/wild type ratio. We performed computer simulations to assess the theoretical relationship between mtDNA staining efficiency and the probability of seeing a segregation unit as a red, green or yellow dot in dependence of its wild type mutant content. The 9 possible wild type/mutant DNA ratios are plotted for a segregation unit with 8 mtDNAs. Considering that in V_3.2 (67% heteroplasmy on average) ∼60 dots are seen per cell that are mostly only green or red and scarcely yellow, we estimated a 3243 padlock/RCA efficiency of ∼5%. This ∼5% efficiency was also inferred from the ‘50% mutation load’ model experiment using the 2031/12252 probe set in which also little co- localization was observed (not shown). We concluded that padlock/RCA is suited for quantitation of single cell mutation loads (see Figure S1), but not for assessment of its genotypic status. Also the padlock/RCA product is densely filled with DNA and relatively large in size, taking up spaces with diameters up to 0.5 µm. Even when a red and a green RCA product would emanate from the same (sub-microscopic) segregation unit, they could physically be repelled.
(PDF)
