# Supplementary material for: Non-Random mtDNA Segregation Patterns Indicate a Metastable Heteroplasmic Segregation Unit in m.3243A>G Cybrid Cells
Source: PLoS One. 2012 Dec 18;7(12):e52080. doi: 10.1371/journal.pone.0052080 (PMC3525564; doi:10.1371/journal.pone.0052080)

Figure S1. Accuracy of Padlock/RCA

(A) To assess accuracy of Padlock/RCA, a model system experiment was performed in which we simulated 50% heteroplasmy by hybridizing a bicolour Padlock probe set reporting presence of positions m.2031 and m.12252. The padlock probes for these positions directly flank the *Dra*1 restriction sites at positions m.2049 and m.12270 from which the Padlock/RCA process is initiated. Padlock probe sequences for positions m.2031 and m.12252 are as follows (italics: probe sequence; underlined: reporter sequence; normal: linker sequence)
*TTGTTAGACATGGGG*ATTCCTTTTACGACCTCAATGCACATGTTTGGCTCCTCTTCT*GTTGAGAAAGCCATG*
and
*ATCTTGGACAACCAGC*ATTCCTTTTACGACCTCAATGCTGCTGCTGTACTACTCTTCT*GTTGAACTAAGATTCT*
This probe set was hybridized to cells of passage 1 of clone V_3.2 (V_3.2 P1). Panel (A) shows the 'mutation load' distribution in a frequency histogram with a 5% bin for all cells, for cells with ≥ 20 and ≥ 40 dots/cell, while the accompanying table gives average ± SD for the mutation load and for the number of dots/cell at increasing stringency of the dots/cell number criterion from > 0 (all) up to ≥ 100 dots/cell. With the average point mutation load being very close to the expected 50% with SD of ~7% when sampling cells with ≥ 20 total dots/cell, these data show that with Padlock/RCA similar accuracies as with single cell PCR/RFMT are obtained. Although with the M.3243A>G probe set similar high average dots/cell were obtained with some passages, others gave less. It ranged from 20-88 for all 15 passages analysed with the M.3243A>G probe set, with ~ 2-fold variability among passages of a given clone, indicating that procedural aspects affect Padlock/RCA efficiency. In general M.3243A>G mutation load histograms did not change shape significantly when the stringency of the dots/cell was increased, indicating that heteroplasmy histograms can be sampled from sub-populations with the higher dot number. (B) For a passage that yielded intermediate M.3243A>G average dot numbers (V_3.2 P1; average dot number = 59) this is illustrated by showing the minimal effects of excluding cells with < 20 and < 40 dots/cell on the mutation load histogram and the formal statistics. (C) A similar analysis of the passage with the lowest average dot number in this study (V_3.18 P2; average dot number = 20) illustrates that with such low average total dots/cell numbers, the contribution of cells with > 40 dots/cell reduces to < 10%. To minimize sampling error, we demanded that more than 30% of the cells contribute to the mutation load histograms. (D) Graph showing the percentage contribution of cells with ≥ 20 and ≥ 40 dots/cell as a function of the average dot number per cell. Data from all 15 passages analysed with the M.3243A>G probe set were used. As is evident from the graph, the 'more than 30% of all cells contribution' criterion necessitated for 3 of the 15 passages analysed by M.3243A>G Padlock/RCA use of cells with ≥ 20 dots. In such cases 10% bin histograms were used to present results.

A **V_3.2 P1 with 2031/12252 probe set**

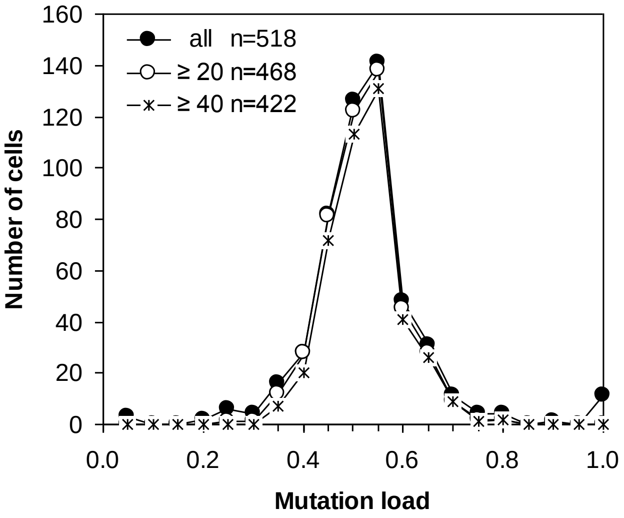

	Heteroplasmy Average (SD)	# Dots/cell Average (SD)	# Cells (%)
all	50.2 (11.9)	91.3 (57.1)	518 (100)
≥ 20	49.7 (7.5)	99.9 (52.9)	468 (90.3)
≥ 40	50.1 (7.1)	107.5 (50.2)	422 (81.5)
≥ 100	50.3 (6.5)	149.6 (40.9)	198 (38.2)

B **V_3.2 P1 with 3243 probe set**

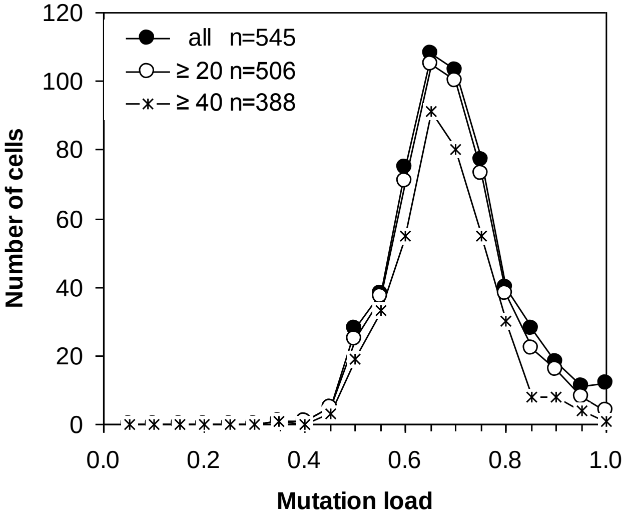

	Heteroplasmy Average (SD)	# Dots/cell Average (SD)	# Cells (%)
all	66.9 (11.4)	59.0 (37.2)	545 (100)
≥ 20	66.0 (10.5)	62.5 (36.2)	506 (92.8)
≥ 40	64.9 (9.5)	72.5 (35.7)	388 (71.2)
≥ 100	62.3 (8.4)	138.4 (49.0)	56 (10.3)

C **V_3.18 P2 with 3243 probe set**

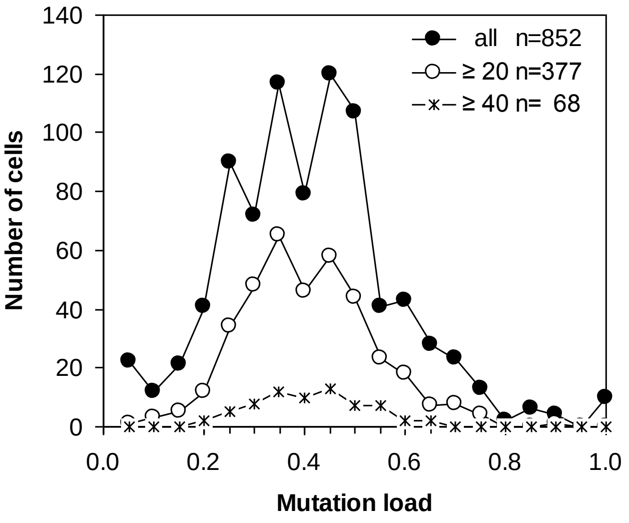

	Heteroplasmy Average (SD)	# Dots/cell Average (SD)	# Cells (%)
all	38.8 (17.4)	20.4 (13.3)	852 (100)
≥ 20	38.2 (12.8)	51.5 (12.7)	377 (44.2)
≥ 40	38.7 (10.4)	50.9 (17.3)	68 (7.9)
≥ 100	40.2 (3.9)	122.6 (10.0)	3 (0.3)

D

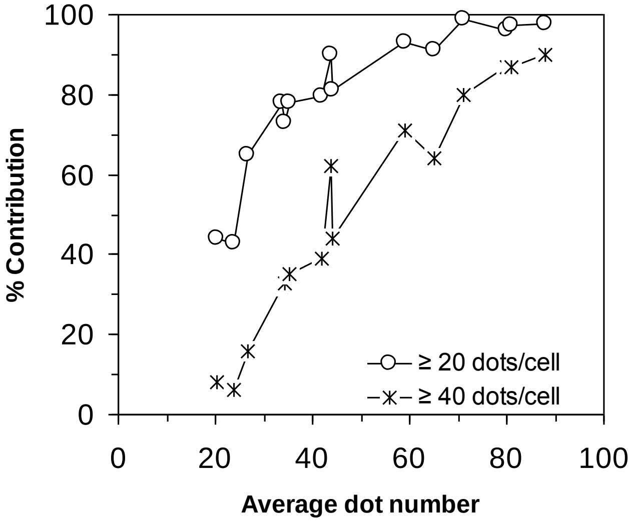

Supplement: Figure S1 — Accuracy of Padlock/RCA. (A) To assess accuracy of Padlock/RCA, a model system experiment was performed in which we simulated 50% heteroplasmy by hybridizing a bicolour Padlock probe set reporting presence of positions m.2031 and m.12252. The padlock probes for these positions directly flank the Dra1 restriction sites at positions m.2049 and m.12270 from which the Padlock/RCA process is initiated. Padlock probe sequences for positions m.2031 and m.12252 are as follows (italics: probe sequence; underlined: reporter sequence; normal: linker sequence) TTGTTAGACATGGGG ATTCCTTTTACGACCTCAATGCACATGTTTGGCTCCTCTTCTGTTGAGAAAGCCATG and ATCTTGGACAACCAGC ATTCCTTTTACGACCTCAATGCTGCTGCTGTACTACTCTTCTGTTGAACTAAGATTCT . This probe set was hybridized to cells of passage 1 of clone V_3.2 (V_3.2 P1). Panel (A) shows the ‘mutation load’ distribution in a frequency histogram with a 5% bin for all cells, for cells with ≥20 and ≥40 dots/cell, while the accompanying table gives average ± SD for the mutation load and for the number of dots/cell at increasing stringency of the dots/cell number criterion from >0 (all) up to ≥100 dots/cell. With the average point mutation load being very close to the expected 50% with SD of ∼7% when sampling cells with ≥20 total dots/cell, these data show that with Padlock/RCA similar accuracies as with single cell PCR/RFMT are obtained. Although with the M.3243A>G probe set similar high average dots/cell were obtained with some passages, others gave less. It ranged from 20–88 for all 15 passages analysed with the M.3243A>G probe set, with ∼2-fold variability among passages of a given clone, indicating that procedural aspects affect Padlock/RCA efficiency. In general M.3243A>G mutation load histograms did not change shape significantly when the stringency of the dots/cell was increased, indicating that heteroplasmy histograms can be sampled from sub-populations with the higher dot number. (B) For a passage that yielded intermediate M.3243A>G average dot numbers ( [file pone.0052080.s001.pdf]
