# Supplementary material for: Non-Random mtDNA Segregation Patterns Indicate a Metastable Heteroplasmic Segregation Unit in m.3243A>G Cybrid Cells
Source: PLoS One. 2012 Dec 18;7(12):e52080. doi: 10.1371/journal.pone.0052080 (PMC3525564; doi:10.1371/journal.pone.0052080)
Supplement: Figure S2 — Efficiency of Padlock/RCA. Earlier FISH work [58], [59] strongly indicated that detection efficiency (i.e. the fraction of target molecules detected) with small probes (<5000 bp) is low and determined largely by accessibility of the target for detection reagents. In Padlock/RCA FISH a series of in situ enzymatic reaction is involved in the detection, viz. restriction enzyme digestion, 5′-3′ exonuclease, ligation, 3′-5′ nuclease and 5′-3 polymerization. Their cumulative efficiencies will negatively affect overall Padlock/RCA FISH efficiency in the formaldehyde-fixed cells used. If one would e.g. for V_3.2 with its ∼60 dots/cell with the 3243 probe set and ∼1800 mtDNA on average per cell consider a dot as originating from single mtDNAs not organized in any structure then efficiency is apparently only ∼3%. However, in the faithful nucleoid model of Jacobs et al [25] as well as in the one proposed here, multiple mtDNAs are presumed to be organized in a limited space. This implies that co-localization of red and green padlock/RCA signals should be seen in heteroplasmic cells, the frequency of which is a function of the mtDNA staining efficiency, the number of mtDNAs per segregation unit and their mutant/wild type ratio. We performed computer simulations to assess the theoretical relationship between mtDNA staining efficiency and the probability of seeing a segregation unit as a red, green or yellow dot in dependence of its wild type mutant content. The 9 possible wild type/mutant DNA ratios are plotted for a segregation unit with 8 mtDNAs. Considering that in V_3.2 (67% heteroplasmy on average) ∼60 dots are seen per cell that are mostly only green or red and scarcely yellow, we estimated a 3243 padlock/RCA efficiency of ∼5%. This ∼5% efficiency was also inferred from the ‘50% mutation load’ model experiment using the 2031/12252 probe set in which also little co- localization was observed (not shown). We concluded that padlock/RCA is suited for quantitation of single cell [file pone.0052080.s002.pdf]

Figure S2. Efficiency of Padlock/RCA

Earlier FISH work [58,59] strongly indicated that detection efficiency (i.e. the fraction of target molecules detected) with small probes (< 5000bp) is low and determined largely by accessibility of the target for detection reagents. In Padlock/RCA FISH a series of in situ enzymatic reaction is involved in the detection, viz. restriction enzyme digestion, 5’-3’ exonuclease, ligation, 3’-5’ nuclease and 5’-3 polymerization. Their cumulative efficiencies will negatively affect overall Padlock/RCA FISH efficiency in the formaldehyde-fixed cells used. If one would e.g. for V_3.2 with its ~ 60 dots/cell with the 3243 probe set and ~1800 mtDNA on average per cell consider a dot as originating from single mtDNAs not organized in any structure then efficiency is apparently only ~ 3%. However, in the faithful nucleoid model of Jacobs *et al* [25] as well as in the one proposed here, multiple mtDNAs are presumed to be organized in a limited space. This implies that co-localization of red and green padlock/RCA signals should be seen in heteroplasmic cells, the frequency of which is a function of the mtDNA staining efficiency, the number of mtDNAs per segregation unit and their mutant/wild type ratio.

We performed computer simulations to assess the theoretical relationship between mtDNA staining efficiency and the probability of seeing a segregation unit as a red, green or yellow dot in dependence of its wild type mutant content. The 9 possible wild type/mutant DNA ratios are plotted for a segregation unit with 8 mtDNAs. Considering that in V_3.2 (67% heteroplasmy on average) ~ 60 dots are seen per cell that are mostly only green or red and scarcely yellow, we estimated a 3243 padlock /RCA efficiency of ~ 5%. This ~ 5% efficiency was also inferred from the ‘50% mutation load’ model experiment using the 2031/12252 probe set in which also little co- localization was observed (not shown). We concluded that padlock/RCA is suited for quantitation of single cell mutation loads (see Figure S1), but not for assessment of its genotypic status. Also the padlock/RCA product is densely filled with DNA and relatively large in size, taking up spaces with diameters up to 0.5 µm. Even when a red and a green RCA product would emanate from the same (sub-microscopic) segregation unit, they could physically be repelled.

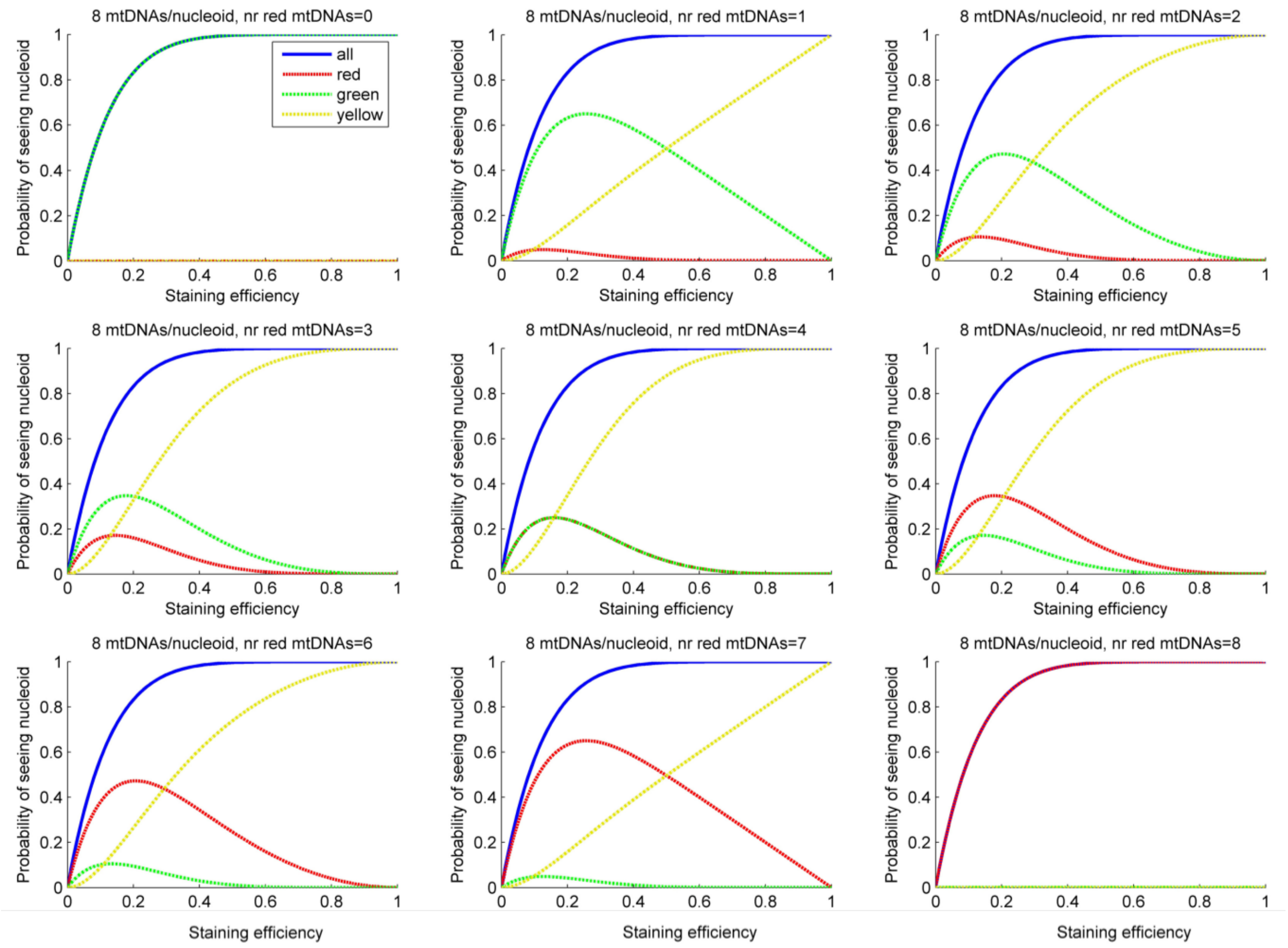

Theoretical relationship between m.3243A>G padlock/RCA mtDNA staining efficiency and the probability of seeing a segregation unit (in this case a hypothetical nucleoid) with 8 mtDNAs as a red, green or yellow dot in dependence of its wild type to mutant content.
